# Burden of disease from inadequate water, sanitation and hygiene for selected adverse health outcomes: An updated analysis with a focus on low- and middle-income countries

**DOI:** 10.1016/j.ijheh.2019.05.004

**Published:** 2019-06

**Authors:** Annette Prüss-Ustün, Jennyfer Wolf, Jamie Bartram, Thomas Clasen, Oliver Cumming, Matthew C. Freeman, Bruce Gordon, Paul R. Hunter, Kate Medlicott, Richard Johnston

**Affiliations:** a Department of Public Health, Environment and Social Determinants of Health, World Health Organization, Geneva, Switzerland; b Gillings School of Global Public Health, University of North Carolina at Chapel Hill, Chapel Hill, NC, USA; c Department of Environmental Health, Rollins School of Public Health, Emory University, Atlanta, GA, USA; d Department of Disease Control, London School of Hygiene and Tropical Medicine, London, UK; e The Norwich School of Medicine, University of East Anglia, Norwich, UK; f Department of Environmental Health, Tshwane University of Technology, Pretoria, South Africa

**Keywords:** Burden of disease, Comparative risk assessment, Drinking water, Water, Sanitation, Hygiene, Diarrhoea, Hand washing, CRA, comparative risk assessment, DALYs, disability-adjusted life years, HICs, high-income countries, JMP, WHO/UNICEF Joint Monitoring Programme for Water Supply, Sanitation and Hygiene, LMICs, low- and middle-income countries, WASH, water, sanitation and hygiene behaviours

## Abstract

**Background:**

To develop updated estimates in response to new exposure and exposure-response data of the burden of diarrhoea, respiratory infections, malnutrition, schistosomiasis, malaria, soil-transmitted helminth infections and trachoma from exposure to inadequate drinking-water, sanitation and hygiene behaviours (WASH) with a focus on low- and middle-income countries.

**Methods:**

For each of the analysed diseases, exposure levels with both sufficient global exposure data for 2016 and a matching exposure-response relationship were combined into population-attributable fractions. Attributable deaths and disability-adjusted life years (DALYs) were estimated for each disease and, for most of the diseases, by country, age and sex group separately for inadequate water, sanitation and hygiene behaviours and for the cluster of risk factors. Uncertainty estimates were computed on the basis of uncertainty surrounding exposure estimates and relative risks.

**Findings:**

An estimated 829,000 WASH-attributable deaths and 49.8 million DALYs occurred from diarrhoeal diseases in 2016, equivalent to 60% of all diarrhoeal deaths. In children under 5 years, 297,000 WASH-attributable diarrhoea deaths occurred, representing 5.3% of all deaths in this age group. If the global disease burden from different diseases and several counterfactual exposure distributions was combined it would amount to 1.6 million deaths, representing 2.8% of all deaths, and 104.6 million DALYs in 2016.

**Conclusions:**

Despite recent declines in attributable mortality, inadequate WASH remains an important determinant of global disease burden, especially among young children. These estimates contribute to global monitoring such as for the Sustainable Development Goal indicator on mortality from inadequate WASH.

## 1 Introduction

Global burden of disease assessments are important to identify priorities for improving population health and tracking changes in the relative importance of different diseases, injuries and risk factors (Murray and Lopez, 2013). The burden of disease from inadequate drinking water, sanitation and hygiene behaviours (WASH) has been estimated at various times in previous decades (Forouzanfar et al., 2016, 2015; Gakidou et al., 2017; Lim et al., 2012; Murray and Lopez, 1996; Prüss-Ustün et al., 2014, 2008; Stanaway et al., 2018; WHO, 2004, 2002); inadequate drinking water as used in this work includes unsafe water and water with insufficient access. While some of these assessments focused on diarrhoeal disease (Forouzanfar et al., 2015; Lim et al., 2012; Murray and Lopez, 1996; Prüss-Ustün et al., 2014; WHO, 2002) others also assessed the WASH-attributable disease burden of other health outcomes such as soil-transmitted helminth infections, malaria, trachoma, schistosomiasis, lymphatic filariasis, lower respiratory infections, and protein energy malnutrition (Forouzanfar et al., 2016; Gakidou et al., 2017; Prüss-Ustün et al., 2008; Stanaway et al., 2018; WHO, 2004). These assessments present very different burden of disease estimates because of differences in methods used, scope of the estimates, and ongoing improvements in WASH in many regions (Clasen et al., 2014).

Despite improvements, inadequate WASH remains a major global risk factor: In 2015, 844 million people lacked a basic drinking water service, i.e., a drinking water source protected from recontamination within 30 min’ round-trip to collect water, and nearly 30% of the global population did not use a safely managed drinking water service—a drinking water source located on premises, available when needed and free from contamination (WHO and UNICEF, 2017). In terms of access to sanitation, 2.3 billion people were lacking a basic sanitation service—an improved sanitation facility that is not shared with other households—and more than 60% were not using a safely managed sanitation service—a sanitation facility that safely disposes excreta in-situ or that ensures that excreta are safely treated off-site (WHO and UNICEF, 2017). Estimates suggest that one in four persons worldwide does not have access to a handwashing facility with soap and water on premises and that only 26% of potential faecal contacts are followed by handwashing with soap (Wolf et al., 2018b). Furthermore, only 45% of the population live in communities in which coverage with basic sanitation services is above 75% (Wolf et al., 2018c).

The objective of this paper is to present updated WASH-attributable burden of diarrhoeal disease estimates for the year 2016 and to add the WASH-attributable burden of further selected adverse health outcomes including respiratory infections, malnutrition, schistosomiasis, malaria, soil-transmitted helminth infections and trachoma. It needs to be acknowledged that – depending on the available evidence - not all estimates are based on the same level of evidence, use different counterfactual exposure distributions and apply different assumptions. To reduce this disease burden from a broad range of diseases, very different intervention strategies would be required which are further outlined below. This paper provides the basis for reporting on Sustainable Development Goal indicator (3.9.2) on WASH-attributable mortality (United Nations, 2018).

## 2 Methods

### 2.1 Framework for estimation

“Inadequate WASH” as used in this article spans a range of WASH services, behaviours and related risks for specific health outcomes, including, amongst others, drinking water, sanitation and hygiene (e.g., diarrhoea, protein-energy malnutrition), and water resources management (e.g., malaria). Sanitation and drinking water services, and presence of a handwashing facility with soap and water on premises are defined following the WHO/UNICEF Joint Monitoring Programme for Water Supply, Sanitation and Hygiene (JMP)(WHO and UNICEF, undated). Table 1 presents a list of adverse health outcomes that can at least partly be attributed to inadequate WASH and whether this relation has previously been quantified. Some of the outcomes from Table 1 for which global WASH-attributable disease burden estimates are available (right column) are not included in this analysis as high quality evidence on the exposure-response relationship is lacking.

**Table 1:** Adverse health outcomes that are at least partly attributable to inadequate water, sanitation and hygiene behaviours.

Global WASH-attributable disease burden not quantified	Global WASH-attributable disease burden estimates available	
Health outcomes	Health outcomes	Main WASH exposure
Arsenicosis Cyanobacterial toxins Fluorosis Hepatitis A, E Lead poisonings Legionellosis Leptospirosis Methaemoglobinaemia Neonatal conditions and maternal outcomes Poliomyelitis Scabies Spinal injury	**Ascariasis**	sanitation
	Cancer (bladder)	drinking water
	Dengue	water resource management/water bodies
	**Diarrhoeal diseases**	drinking water, sanitation, hygiene behaviours*
	Drowning^d^	recreational water/water bodies
	**Hookworm disease**^a^	Sanitation
	Japanese Encephalitis	water resource management/agricultural practices
	Lymphatic filariasis	water resource management/water bodies
	**Malaria**^d^	water resource management/water bodies
	Musculoskeletal diseases	drinking water
	Onchocerciasis	water resource management
	**Protein-energy malnutrition**^a,b,c^	drinking water, sanitation, hygiene behaviours*
	**Respiratory infections**^c^	hygiene behaviours*
	**Schistosomiasis**^a,b,c,d^	drinking water, sanitation, hygiene behaviours*, water resource management/agricultural practices/recreational water
	**Trachoma**^**a,**c^	sanitation, hygiene behaviours*
	**Trichuriasis**^a^	Sanitation

The listed diseases are based on prior work (Prüss-Ustün et al., 2016, 2008). Health outcomes quantified in this article are written in bold. *hygiene behaviours include hand hygiene(diarrhoeal diseases, protein-energy malnutrition, trachoma), face hygiene (trachoma), food hygiene (hookworm) and bathing (schistosomiasis).

This disease burden assessment for the year 2016 preferably includes adverse health outcomes for which the WASH-attributable fraction of disease burden can be estimated using comparative risk assessment (CRA, respective diseases are diarrhoea, ARI and schistosomiasis). CRAs are based on detailed, i.e., by level of exposure, age group and sex, exposure and exposure-response information (Ezzati et al., 2002; WHO, 2004). In addition, we present WASH-attributable disease burden estimates from other health outcomes for which sufficient exposure and exposure-response data was available but which are based on weaker evidence, more assumptions and different counterfactual exposure distributions (malnutrition, malaria, soil-transmitted helminth infections and trachoma). WASH-attributable burden of disease estimates were calculated for 132 low- and middle-income countries as the available epidemiological evidence originates mainly from these settings. For diarrhoea (only for hygiene as risk factor) and acute respiratory infections, estimates were calculated for 183 low-, middle- and high-income countries. Countries are WHO Member States with income levels defined by the World Bank for 2016 (World Bank, 2016) which were grouped into the six WHO Regions (Sub-Saharan Africa, America, Eastern Mediterranean, Europe, South-East Asia, and Western Pacific (WHO, 2017a)). Data on total deaths and disability-adjusted life years (DALYs) by disease or condition were taken from the WHO Global Health Observatory for the year 2016 (WHO, 2018a)**.** These data are publicly available and can be assessed from the following website (WHO, 2018b).

### 2.2 Population attributable fractions of disease for individual risk factors and for the cluster of risks

Disease burden attributable to a risk factor is estimated using the population attributable fraction (PAF) which is the proportion of disease or death that could be prevented if exposures were reduced to an alternative or counterfactual scenario, while other conditions remain unchanged (Ezzati et al., 2002; WHO, 2004). The calculation of the PAF requires the proportion of the population exposed to the different levels of the risk factor and the corresponding exposure-response relationship (Vander Hoorn et al., 2004):

(1) $$PAF=\;\frac{\sum_{j=1}^{n}p_{j}\;\left(RR_{j}-1\right)}{\sum_{j=1}^{n}p_{j}\left(RR_{j}-1\right)+1}$$

where $p_{j}$ is the proportion of the population exposed at exposure level j, $RR_{j}$ is the relative risk at exposure level j and *n* is the total number of exposure levels.

Exposure levels of drinking water, sanitation and hygiene are related by similar mechanisms and policy interventions. The following formula has been proposed for the estimation of burden attributable to a interlinked cluster of risk factors (Lim et al., 2012) (relevant for the diarrhoea and schistosomiasis burden):

(2) $$PAF=1-\;\overset{R}{\underset{r=1}{\prod }}\left(1-PAF_{r}\right)$$

where *r* is the individual risk factor, and *R* the total number of risk factors accounted for in the cluster.

### 2.3 Choice of counterfactual exposure levels for WASH-attributable disease burden estimation

The counterfactual exposure distribution can be defined in various ways including the theoretical, the plausible, the feasible and the cost-effective minimum risk exposure distributions (Murray et al., 2003). The theoretical minimum risk exposure distribution refers to the exposure level with the lowest population health risk, irrespective of whether this level is currently attainable in practice. The plausible minimum risk exposure distribution refers to a level which is imaginable without necessarily being likely or feasible in the near future. The feasible minimum risk exposure distribution is a level that has been observed in some population and the cost-effective minimum risk exposure distribution considers the costs of exposure reduction for choosing the alternative exposure scenario (Murray et al., 2003).

Depending on the type and quality of the available evidence, we chose different definitions of the counterfactual exposure distribution for the various adverse health outcomes included in this analysis (Table 2). For WASH-attributable diarrhoeal disease burden estimation, we applied the plausible minimum risk exposure distribution which includes that all the population boils and filters their drinking water and prevents recontamination, lives in a community in which coverage with basic sanitation services exceeds 75% and practices handwashing with soap after potential faecal contact. The WASH-attributable burden of malnutrition estimates are based on the diarrhoea estimates using a pooled analysis of the fraction of stunting attributable to repeated diarrhoea episodes (Checkley et al., 2008). We also used the plausible minimum risk exposure distribution for the hygiene-attributable disease burden of acute respiratory infections. For trachoma and soil-transmitted helminth infections, we used the theoretical minimum risk exposure distribution and assume that the burden of these diseases could be completely prevented through adequate WASH, based on current knowledge on disease transmission which basically occurs through inadequate sanitation and hygiene. The theoretical minimum risk exposure distribution is approximated here as all the population using safely managed drinking water, i.e., a basic drinking water service accessible on premises, available when needed and free from contamination, safely managed sanitation, i.e., a basic sanitation service that safely disposes excreta in-situ or that ensures that excreta are safely treated off-site, and all the population having access to essential hygiene conditions and performing essential hygiene practices that help maintain health and prevent the spread of disease, including hand- and facewashing, menstrual hygiene management and food hygiene (WHO and UNICEF, undated). Also for the WASH-attributable malaria burden estimates, we used the theoretical minimum risk exposure distribution of all the population being exposed to safe water resource management for which a corresponding exposure-response relationship from meta-analysis is available (Keiser et al., 2005). For the WASH-attributable schistosomiasis disease burden estimation, the applied counterfactual is equivalent to a feasible minimum risk exposure distribution which is access to basic drinking water and sanitation services. This is again due to the available matching exposure-response relationships for these exposures (Freeman et al., 2017; Grimes et al., 2014).

**Table 2:** Information on counterfactual, outcome association and potential for bias by health outcome.

health outcome	WASH counterfactual exposure definition	prevalence of WASH counterfactual exposure in 2016	RR for/association between WASH counterfactual exposure and outcome# (against lowest level of exposure, e.g., unimproved WASH)	counterfactual definition used	potential for bias
diarrhoea	water: household water treatment using filtering or boiling	33.1% (WHO and UNICEF, undated)	RR 0.52 (0.35, 0.77)* (Wolf et al., 2018a)	plausible minimum risk	predominately non-blinded intervention studies but bias-adjustment performed
	sanitation: basic sanitation in a community >75% sanitation coverage	45.3% (Wolf et al., 2018c)	RR 0.55 (0.34, 0.91) (Wolf et al., 2018a)		
	hygiene: handwashing with soap after potential faecal contact	26.2% (Wolf et al., 2018b)	RR 0.86 (0.35, 2.07)* (Wolf et al., 2018a)		
acute respiratory infections	hygiene: handwashing with soap after potential faecal contact	26.2% (Wolf et al., 2018b)	RR 0.84 (0.79, 0.89) (Rabie and Curtis, 2006)	plausible minimum risk	predominantly non-blinded intervention studies
protein-energy malnutrition	*same as for diarrhoea*	*same as for diarrhoea*	combining the PAF for stunting attributable to diarrhoea (25% (8%, 38%)) (Checkley et al., 2008) with the PAF of WASH-attributable diarrhoeal disease (60% (54%, 65%))	*same as for diarrhoea*	includes only WASH-attributable burden via diarrhoea, only stunting is considered as indicator for malnutrition
schistosomiasis	basic drinking water and basic sanitation services	basic drinking water: 87.2%; basic sanitation: 62.0% (WHO and UNICEF, undated)	basic drinking water: RR 0.53 (0.47, 0.61) (Grimes et al., 2014); basic sanitation: RR 0.65 (0.54, 0.78) (Freeman et al., 2017)	feasible minimum risk	RR estimates from observational studies only
malaria	safe water resource management	0% (Keiser et al., 2005)	RR 0.21 (0.13–0.33) (Keiser et al., 2005)	theoretical minimum risk	disease burden estimates based on stronger assumptions
soil-transmitted helminth infections	safely managed water and safely managed sanitation services, essential hygiene conditions and essential hygiene practices	NA	RR 0	theoretical minimum risk	disease burden estimates based on stronger assumptions
trachoma	safely managed water and safely managed sanitation services, essential hygiene conditions and essential hygiene practices	NA	RR 0	theoretical minimum risk	disease burden estimates based on stronger assumptions

RR: relative risk, NA: not applicable, # separate RR for water, sanitation and hygiene are combined using equation (2), * adjusted for potential non-blinding bias.

### 2.4 Estimation of burden of disease attributable to inadequate WASH

The burden of disease attributable to each risk factor (AB), or to the cluster of risk factors, in deaths or DALYs, was obtained by multiplying the PAF by the total burden of each respective disease (B):

(3) AB = PAF x B

The PAFs were applied equally to burden of disease in deaths and DALYs and we assumed that the WASH-attributable case fatality was the same as the mean case fatality of the respective diseases.

### 2.5 Uncertainty estimates

To estimate uncertainty intervals, we developed a Monte Carlo simulation of the results with 5000 draws of the exposure distribution, and of the relative risks. As lower and upper uncertainty estimates we used the 2.5 and 97.5 percentiles of the PAFs, attributable deaths and DALYs resulting from the Monte Carlo analysis. Uncertainty estimates were calculated using @RISK-software, version 6 (@RISK, n.d.).

We are following guidelines for accurate and transparent health estimates reporting (GATHER)(“GATHER: Guidelines for Accurate and Transparent Health Estimates Reporting,” n.d.; Stevens et al., 2016) and have included a GATHER-checklist as a Supplementary File (S3).

### 2.6 The WASH-attributable burden of diarrhoeal disease

#### 2.6.1 Adjustment for non-blinding bias of interventions for exposure-response estimation

Open trials – that is where participants are not blinded to their allocation – which use subjective outcome measures, such as self-reported diarrhoea, are at high risk of bias (Savović et al., 2012; Wood et al., 2008). Exposure-response relationships linking point-of-use drinking water or hygiene interventions and diarrhoea were therefore bias-adjusted based on empirical evidence (Savović et al., 2012)(Tables S1 and S2 in the Supplementary File 1) using a previously published method (Wolf et al., 2018a, 2014). These two types of WASH interventions were chosen for bias adjustment as these interventions usually aim exclusively to improve health which is apparent to the recipient. A detailed description of this approach can also be found in the Supplementary File S1. We present WASH-attributable diarrhoeal disease burden as bias-adjusted estimates in the main text and additionally as non-adjusted estimates in the Supplementary File S1, Tables S3–S5, to show the magnitude of this adjustment and for comparability with other burden of disease assessments.

#### Drinking water

**Fig. 1:**
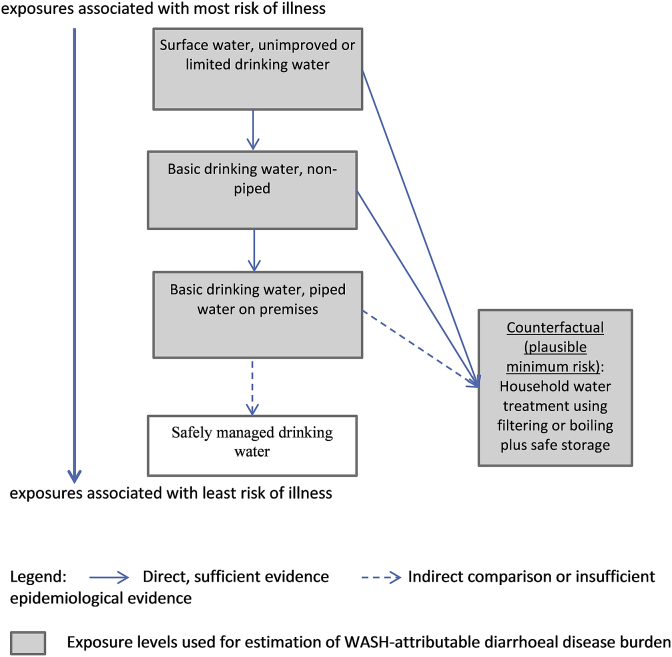
Exposure levels for drinking water-related burden of diarrhoeal disease estimates. Note: these exposure levels are used for the WASH-attributable burden of diarrhoeal disease assessment, exposure levels used for the assessment of other diseases vary. “limited”, “unimproved” and “basic” facilities and services follow definitions of the WHO/UNICEF Joint Monitoring Programme for Water Supply, Sanitation and Hygiene (JMP) (WHO and UNICEF, undated). “Counterfactual” signifies the counterfactual exposure distribution used for the diarrhoeal disease assessment and presents the plausible minimum exposure distribution. The theoretical minimum risk exposure distribution (which is not used for this analysis) would be “safely managed drinking water”. The length of the different arrows in not intended to quantify differences in disease risk.

Fig. 1 shows drinking water exposure levels and Tables 2 and S1 (Supplementary File 1) show matching exposure-response relationships used for WASH-attributable burden of diarrhoeal disease estimation.

*Exposure estimates*: Data on the relevant exposure levels was available through country-representative household surveys and censuses reported by the JMP (WHO and UNICEF, undated). Estimates for the year 2016 were derived using multilevel modeling (Wolf et al., 2013) of about 1400 data points for each of the different categories of drinking water supply and about 130 data points for each of the different categories of household water treatment. Exposure estimates for the different levels of drinking water relevant for burden of disease calculation are available by country as a Supplementary File (S2).

*Exposure-response relationship*: As the evidence on additional improvements – such as improvements in water quality and availability - on piped water to premises remains limited, we chose household water filtering or boiling with prevention of recontamination as the counterfactual exposure level. Corresponding exposure-response relationships were taken from the most recent meta-analysis (Wolf et al., 2018a). (Tables 2 and S1 in the Supplementary File 1)

#### Sanitation

**Fig. 2:**
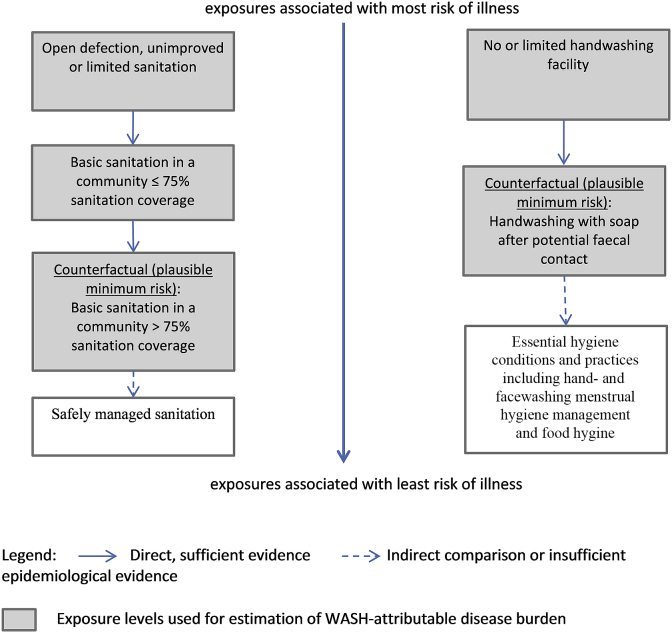
Exposure levels for sanitation-related (left) and hygiene-related (right) burden of disease estimates. Note: these exposure levels are used for the WASH-attributable burden of diarrhoeal disease and – for hygiene - acute respiratory infections assessment, exposure levels used for burden of disease estimation of other diseases vary. “limited”, “unimproved” and “basic” facilities and services follow definitions of the WHO/UNICEF Joint Monitoring Programme for Water Supply, Sanitation and Hygiene (JMP) (WHO and UNICEF, undated). “Counterfactual” signifies the counterfactual exposure distribution used for the diarrhoeal disease and respiratory infections assessment and presents the plausible minimum exposure distribution. The theoretical minimum risk exposure distribution (which is not used for the diarrhoea and respiratory infections analysis) would be “Safely managed sanitation” and “Essential hygiene conditions and practices including hand- and facewashing, menstrual hygiene management and food hygiene”. The length of the different arrows in not intended to quantify differences in disease risk.

Fig. 2 shows sanitation exposure levels. Tables 2 and S2 (Supplementary File 1) shows the matching exposure-response relationship used for WASH-attributable burden of diarrhoeal disease estimation.

*Exposure estimates*: Sanitation exposure data was available from the JMP (WHO and UNICEF, undated). Exposure estimates of access to basic sanitation services in a community with greater than 75% coverage with basic sanitation services is based on an analysis of survey data at cluster-level (Wolf et al., 2018c). Exposure estimates for the different levels of sanitation relevant for burden of disease calculation are available by country as a Supplementary File (S2).

*Exposure-response relationship*: New evidence has recently emerged on additional benefits on diarrhoeal disease from safe sanitation when people live in communities with high sanitation coverage (e.g., (Fuller and Eisenberg, 2016; Jung et al., 2017b, 2017a)). This has led to using basic sanitation services in a community in which more than 75% of people are covered with basic sanitation services as the counterfactual exposure scenario. The choice of the cut-off at 75% sanitation coverage is based on prior sanitation intervention studies which found increased diarrhoea reductions after that point (Wolf et al., 2018c, 2018a).

As a sensitivity analysis, we included the recently published results of four WASH intervention studies (Humphrey et al., 2019; Luby et al., 2018; Null et al., 2018; Reese et al., 2018) in the calculation of the exposure-response relationship between inadequate sanitation and diarrhoeal disease. Results of these studies had not been published at the time of the systematic review and meta-analysis that provided the exposure-response relationships for this burden of disease assessment (Wolf et al., 2018a).

#### Hygiene

Fig. 2 shows hygiene exposure levels and Tables 2 and S2 (Supplementary File 1) show matching exposure-response relationships used for burden of disease estimation.

*Exposure estimates*: Exposure estimates are based on “having a handwashing facility with soap and water on premises”, i.e., a basic handwashing facility (WHO and UNICEF, 2018a), and are available through country-representative household surveys such as Demographic Health Surveys and Multiple Indicator Cluster Surveys through the JMP (WHO and UNICEF, undated). Because access to a basic handwashing facility would overestimate actual handwashing practices, this proxy indicator has been converted to actual handwashing with soap prevalence based on an analysis of the association between presence of a basic handwashing facility and observed handwashing with soap (Wolf et al., 2018b). Exposure estimates for handwashing with soap after potential faecal contact are available by country as a Supplementary File (S2).

*Exposure-response relationship*: The relative risk from a recent systematic review and meta-analysis of WASH intervention studies and diarrhoeal disease (Wolf et al., 2018a) associated with the sub-group of studies focusing on “handwashing promotion” matched best the exposure and was therefore taken for burden of disease calculation.

### 2.7 The WASH-attributable burden of further selected health outcomes

#### 2.7.1 Acute respiratory infections

Hands act frequently as carriers for respiratory pathogens which can enter the body via hand-to-face contact (Warren-Gash et al., 2013). In addition, some forms of respiratory viral disease are transmitted via the faecal-oral route (Rabie and Curtis, 2006).

*Exposure estimates:* Only inappropriate hygiene is considered as risk factor for acute respiratory infections. The same hygiene exposure data as for the analysis of the WASH-attributable diarrhoeal disease burden were taken (handwashing with soap after potential faecal contact derived from access to a handwashing facility with soap and water (Wolf et al., 2018b)).

*Exposure-response relationship*: The relative risk of 0.84 for washing hands with soap and respiratory infections is based on a meta-analysis of seven intervention studies in high-income countries (HICs) (Rabie and Curtis, 2006) which is similar to a more recent pooled estimate from low- and middle-income countries (LMICs) based on only three studies (Mbakaya et al., 2017). Only one of the seven hand-hygiene intervention studies was blinded and used a placebo hand-sanitizer in the control group (White et al., 2001).

#### 2.7.2 Protein-energy malnutrition

Inadequate WASH can be linked to nutritional status via diarrhoea, environmental enteropathy, (subclinical) enteropathogen infections and soil-transmitted helminth infections (Dangour et al., 2013; MAL-ED Network Investigators, 2017; Schnee et al., 2018).

*Exposure estimates*: As the WASH-attributable malnutrition estimates are based on the WASH-attributable diarrhoea estimates, the same exposure levels are used as for the WASH-attributable diarrhoeal disease burden estimation.

*Exposure-response relationship*: A pooled analysis of nine prospective datasets from five countries estimated that 25% of stunting could be attributed to repeated diarrhoea episodes in children (Checkley et al., 2008). This estimate is combined with the fraction of WASH-attributable diarrhoeal disease burden in children under five to estimate the fraction of the WASH-attributable malnutrition burden.

As a sensitivity analysis, disease burden of protein-energy malnutrition was calculated using diarrhoea estimates that were not adjusted for non-blinding bias.

#### 2.7.3 Schistosomiasis

Schistosomiasis can occur when people contact water containing certain aquatic snails that have been infested with parasitic worms; these worms have a human life cycle and are discharged through human excreta (WHO, 2018c).

*Exposure estimates*: The relevant exposure levels for the analysis of the WASH-attributable schistosomiasis burden were use of basic drinking water and sanitation services and surface, unimproved or limited drinking water and open defecation, unimproved or limited sanitation. Data on these exposures were available through the JMP (WHO and UNICEF, undated) with estimates derived for 2016 as described for diarrhoea (Wolf et al., 2013)(Supplementary File S2).

*Exposure-response relationship*: The pooled relative risk from meta-analysis of 0.53 (0.47, 0.61) links access to basic drinking water services versus surface, unimproved or limited drinking water (Grimes et al., 2014). The pooled relative risk of 0.65 (0.54, 0.78) for sanitation links basic sanitation services and open defecation, unimproved or limited sanitation and is the mean relative risk combining the association between sanitation and *Schistosoma mansoni* and *S. haematobium* weighted by the precision of the estimates (Freeman et al., 2017). These relative risks include data from observational studies only (cross-sectional and case-control design).

As a sensitivity analysis we calculated the WASH-attributable schistosomiasis burden using a population attributable fraction (PAF) of 82% as previously estimated through an expert survey (Prüss-Ustün et al., 2016). This 82% relates to the fraction of schistosomiasis that was assumed to be preventable through adequate WASH while it was acknowledged that probably 100% of schistosomiasis burden could be attributed to environmental risks (Prüss-Ustün et al., 2016).

#### 2.7.4 Malaria

Environmental management in malaria prevention often includes water resource management - for example, the installation, cleaning and maintenance of drains, the systematic elimination of standing water pools, the siting of settlements away from vector breeding sites (dry-belting) - but also measures applied to the human habitat such as mosquito-proofing of houses (Keiser et al., 2005).

*Exposure estimates*: Globally, very limited water resource management have been undertaken and environmental management interventions almost disappeared when dichlorodiethyltrichloroethane (DDT) appeared (Keiser et al., 2005). Therefore the relevant exposure levels are universally implemented safe water resource management as theoretical minimum risk exposure distribution versus no safe water resource management.

Exposure-response relationship: The exposure-response relationship is taken from a meta-analysis of the relation between environmental management and malaria occurrence (Keiser et al., 2005). We chose the more conservative – in terms of the size of the relative risk estimate - approach which was based on stronger evidence, and selected an exposure-response relationship (risk ratio) of 0.21 (0.13–0.33) for modification of human habitation – as compared to 0.12 (0.08, 0.18) for environmental modification.

As a sensitivity analysis we calculated the WASH-attributable malaria burden using previously estimated regional PAFs that were based on expert opinion (Prüss-Ustün et al., 2016).

#### 2.7.5 Soil-transmitted helminth infections

This assessment includes the most predominant soil-transmitted helminths – *Ascaris lumbricoides*, *Trichuris trichiura* and the hookworms. Transmission occurs uniquely through the release of nematode eggs in human excreta from infected individuals into the environment. After the release from the human body, the eggs need to mature for about three weeks to become infective. Susceptible individuals are infected via ingestion of these eggs or penetration of their skin by, or direct ingestion of, the larvae. Also re-infection only occurs due to contact with infective stages in the environment (WHO, 2018d). It is therefore assumed that infections with soil-transmitted helminths would completely cease in case the theoretical minimum exposure level – universal use of safely managed water and safely managed sanitation services, universal access to essential hygiene conditions and universal practice of essential hygiene - would be achieved. The total disease burden from infections with soil-transmitted helminths was therefore entirely attributed to inadequate WASH (Prüss-Ustün et al., 2016).

#### 2.7.6 Trachoma

Trachoma is transmitted via personal contact (e.g., via hands and clothes) and by flies that have been in contact with the discharge of the eyes or the nose of an infected person (WHO, 2018e). It is assumed that through safe disposal of faeces and especially hygiene (face- and handwashing and cleaning of clothes) transmission of trachoma would cease which is also supported through historical evidence (Hu et al., 2010; Mohammadpour et al., 2016). The overall disease burden from trachoma was therefore assumed to be fully attributable to inadequate WASH (Prüss-Ustün et al., 2016). For trachoma, we used the same theoretical minimum exposure level as for soil-transmitted helminths of universal safely managed drinking water, safely managed sanitation, essential hygiene conditions and hygiene practices.

## 3 Results

### 3.1 Exposure estimates

The relevant exposures for WASH-attributable disease burden estimation include access to services and WASH-related behaviours. Water resource management is the relevant exposure for WASH-attributable burden of malaria estimation. In LMICs, 58% of the population used piped water on premises; 30% used a non-piped basic water service; and 13% used surface, unimproved or limited drinking water in 2016 (Table 3). 33% of the population reported boiling or filtering their water. In LMICs, 62% used basic sanitation services and 45% of the population lived in communities with basic sanitation coverage above 75% (Table 4). Worldwide, 74% of the population had access to a basic handwashing facility, 70% in LMICs and 95% in HICs. This resulted in 26% of the global population, 22% in LMICs and 51% in HICs, washing hands with soap after potential faecal contact (Table 5).

**Table 3:** Distribution of the population to exposure levels of drinking water, by region, for 2016.

Region	Percentage of population using						Total
	piped water on premises		basic drinking water, not piped on premises		surface, unimproved or limited water		
	not filtered or boiled^a^	filtered or boiled	not filtered or boiled	filtered or boiled	not filtered or boiled	filtered or boiled	
Sub-Saharan Africa, LMICs	25.5	3.1	29.6	2.0	35.8	4.0	100
America, LMICs	58.3	32.3	4.6	1.1	2.9	0.8	100
Eastern Mediterranean, LMICs	53.8	4.8	26.0	0.7	13.7	0.9	100
Europe, LMICs	55.6	29.3	6.9	4.1	2.5	1.7	100
South-East Asia, LMICs	24.9	12.7	38.6	13.0	7.2	3.5	100
Western Pacific, LMICs	28.5	50.7	8.8	8.3	1.6	2.1	100
Total LMICs	34.1	23.5	22.6	7.0	10.2	2.6	100

a Filtering or boiling means point-of-use water treatment at household-level. The total may not equal the sum of numbers displayed in the rows due to rounding. LMICs: low- and middle-income countries.

**Table 4:** Distribution of the population to exposure levels of sanitation, by region, for 2016.

Region	Percentage of population	
	using basic sanitation services	living in communities with >75% basic sanitation coverage
Sub-Saharan Africa, LMICs	30.8	13.3
America, LMICs	85.1	75.8
Eastern Mediterranean, LMICs	69.1	54.8
Europe, LMICs	92.5	93.3
South-East Asia, LMICs	50.9	31.9
Western Pacific, LMICs	75.1	63.2
Total LMICs	62.0	45.3

LMICs: low and middle income countries.

**Table 5:** Distribution of the population to exposure levels of hygiene, by region, for 2016.

Region	Percentage of population washing hands with soap after potential faecal contact
Sub-Saharan Africa, all	8.4
America, LMICs	36.2
Eastern Mediterranean, LMIC	21.6
Europe, LMICs	24.9
South-East Asia, all	27.8
Western Pacific, LMICs	17.1
Total	26.3
Total HICs	50.6
Total LMICs	21.8

LMICs: low and middle income countries, HICs: high income countries.

### 3.2 Estimates of the WASH-attributable burden of diarrhoeal disease

The total number of diarrhoeal deaths in 2016 was 1.4 million (WHO, 2018f). Of those, 485,000 deaths were attributable to inadequate water, 432,000 to inadequate sanitation and 165,000 to inadequate hygiene behaviours after adjusting for the likely effect of non-blinding bias (Table 6, Table 7, Table 8, Table 9). Inadequate WASH together caused 829,000 diarrhoeal deaths which correspond to about 60% of total diarrhoeal deaths in 2016 that would have been preventable through improving drinking water and sanitation services and handwashing with soap.

**Table 6:** Diarrhoea burden attributable to inadequate water by region, 2016

Region	PAF	(95% CI)	Deaths	(95% CI)	DALYs (in 1 000s)	(95% CI)
Sub-Saharan Africa, LMICs	0.40	(0.22–0.51)	259,073	(140,144–330,643)	16,837	(9120–21,472)
America, LMICs	0.27	(0.02–0.42)	6246	(480–9469)	506	(22–776)
Eastern Mediterranean, LMICs	0.39	(0.19–0.50)	48,947	(24,067–63,413)	3675	(1778–4764)
Europe, LMICs	0.20	(0.02–0.31)	959	(86–1500)	137	(2–215)
South-East Asia, LMICs	0.31	(0.12–0.43)	163,760	(64,307–225,941)	7798	(3067–10,750)
Western Pacific, LMICs	0.21	(0.08–0.30)	5756	(2069–8320)	493	(160–725)
Total LMICs	0.36	(0.19–0.47)	484,741	(231,153–639,285)	29,446	(14,149–38,702)

DALYs: disability-adjusted life years, PAF: population-attributable fraction; LMICs: low- and middle-income countries; for the analysis of burden of diarrhoeal disease attributed to inadequate water the counterfactual exposure distribution (plausible minimum risk) of filtering/boiling of water from any water source with subsequent safe storage was compared to the actual exposure distribution for 2016.

**Table 7:** Diarrhoea burden attributable to inadequate sanitation by region, 2016

Region	PAF	(95% CI)	Deaths	(95% CI)	DALYs (in 1 000s)	(95% CI)
Sub-Saharan Africa, LMICs	0.37	(0.36–0.38)	236,134	(229,625–241,875)	15,303	(14,866–15,684)
America, LMICs	0.14	(0.13–0.16)	3261	(2949–3529)	257	(229–280)
Eastern Mediterranean, LMICs	0.27	(0.24–0.30)	34,425	(30,473–37,781)	2538	(2260–2775)
Europe, LMICs	0.03	(0.02–0.03)	134	(91–161)	20	(14–24)
South-East Asia, LMICs	0.29	(0.25–0.33)	152,986	(129,778–173,011)	7245	(6131–8208)
Western Pacific, LMICs	0.17	(0.15–0.20)	4780	(4041–5413)	403	(332–464)
Total LMICs	0.32	(0.30–0.34)	431,720	(407,090–452,623)	25,765	(24,519–26,825)

DALYs: disability-adjusted life years, PAF: population-attributable fraction; LMICs: low- and middle-income countries; for the analysis of burden of diarrhoeal disease attributed to inadequate sanitation the counterfactual exposure distribution (plausible minimum risk) of having access to basic sanitation in a community with >75% coverage with basic sanitation facilities was compared to the actual exposure distribution for 2016.

**Table 8:** Diarrhoea burden attributable to inadequate hygiene behaviours by region, 2016

Region	PAF	(95% CI)		Deaths	(95% CI)	DALYs (in 1 000s)	(95% CI)
Sub-Saharan Africa, all	0.13	(0–0.61)	85,166		(0–394,782)	5516	(0–25,622)
America, LMICs	0.10	(0–0.47)	2227		(0–10,741)	183	(0–886)
America, HICs	0.08	(0–0.41)	930		(0–4967)	25	(0–131)
Eastern Mediterranean, LMICs	0.12	(0–0.57)	15,013		(0–72,270)	1130	(0–5440)
Eastern Mediterranean, HICs	0.08	(0–0.41)	34		(0–186)	5	(0–27)
Europe, LMICs	0.11	(0–0.54)	537		(0–2605)	72	(0–352)
Europe, HICs	0.08	(0–0.40)	1216		(0–6371)	29	(0–151)
South-East Asia, all	0.11	(0–0.50)	56,419		(0–264,975)	2656	(0–12,477)
Western Pacific, LMICs	0.12	(0–0.55)	3347		(0–15,182)	298	(0–1350)
Western Pacific, HICs	0.08	(0–0.40)	310		(0–1645)	6	(0–31)
Total	0.12	(0–0.56)	165,200		(0–780,443)	9919	(0–46,598)

DALYs: disability-adjusted life years, PAF: population-attributable fraction; LMICs: low- and middle-income countries, HICs: high-income countries; for the analysis of burden of diarrhoeal disease attributed to inadequate hygiene behaviours the counterfactual exposure distribution (plausible minimum risk) of handwashing with soap after potential faecal contact was compared to the actual exposure distribution for 2016.

**Table 9:** Diarrhoea burden attributable to the cluster of inadequate water, sanitation and hygiene behaviours by region, 2016

Region	PAF	(95% CI)	Deaths	(95% CI)	DALYs (in 1 000s)	(95% CI)
Sub-Saharan Africa, all	0.67	(0.62–0.72)	431,700	(398,398–462,156)	27,997	(25,822–29,968)
America, LMICs	0.43	(0.35–0.51)	9861	(8050–11,623)	799	(639–952)
America, HICs	0.08	(0.00–0.25)	930	(0–4967)	25	(0–131)
Eastern Mediterranean, LMICs	0.60	(0.50–0.70)	76,387	(62,928–87,982)	5718	(4787–6531)
Eastern Mediterranean, HICs	0.08	(0.00–0.25)	34	(0–186)	5	(0–27)
Europe, LMICs	0.31	(0.22–0.39)	1481	(1053–1899)	207	(148–265)
Europe, HICs	0.08	(0.00–0.17)	1216	(0–6371)	29	(0–151)
South-East Asia, all	0.56	(0.43–0.68)	295,070	(225,467–356,569)	13,981	(10,634–16,948)
Western Pacific, LMICs	0.43	(0.32–0.53)	11,661	(8651–14,501)	1008	(715–1282)
Western Pacific, HICs	0.08	(0.00–0.23)	310	(0–1645)	6	(0–31)
Total	0.60	(0.54–0.65)	828,651	(753,021–901,072)	49,774	(45,835–53,596)

DALYs: disability-adjusted life years, PAF: population-attributable fraction; LMICs: low- and middle-income countries, HICs: high-income countries.

In children under five years of age, 477,000 diarrhoeal deaths occurred in 2016. Of those 297,000 or 62.2% (adjusted for non-blinding bias) were attributable to inadequate WASH.

Not adjusting the disease burden estimates for non-blinding bias resulted in a total of 1,025,000 deaths which correspond to 74% of total diarrhoeal deaths and 1.8% of all deaths being attributable to inadequate WASH in 2016 (Supplementary File S1, Tables S3–S5).

Inclusion of the results of four additional WASH interventions (Humphrey et al., 2019; Luby et al., 2018; Null et al., 2018; Reese et al., 2018) published after we conducted the systematic review and meta-analysis on WASH interventions and diarrhoeal disease (Wolf et al., 2018a), changed the exposure-response relationship for basic sanitation in low-coverage communities to 0.82 (0.63, 1.06) and in high coverage communities to 0.58 (0.40, 0.84) as compared to 0.76 (0.51, 1.13) and 0.55 (0.34, 0.91) for low- and high-coverage communities respectively without these four studies (Tables 2 and S2 in the Supplementary File 1). This resulted in a reduction of diarrhoeal deaths attributable to inadequate sanitation from 432,000 to 396,000.

### 3.3 Estimates of the WASH-attributable burden of other adverse health outcomes

#### 3.3.1 Acute respiratory infections

Thirteen percent of the overall disease burden of acute respiratory infections was attributable to inadequate handwashing with soap which amounted to 370,000 deaths in 2016 (Table 10). WASH-attributable disease burden from acute respiratory infections by region is given in Table S6 in the Supplementary File 1.

**Table 10:** Summary of WASH-attributable disease burden, 2016

disease	PAF	95% CI	method for PAF estimation	counterfactual exposure level	deaths	DALYs
Schistosomiasis	0.43	0.40–0.46	CRA	feasible minimum risk (universal access to/use of basic water and sanitation services)	10,405	1,095,658
**total WASH-attributable disease burden using a feasible minimum risk**					**10,405**	**1,095,658**
Diarrhoea	0.60*	0.54–0.65	CRA	plausible minimum risk (universal filtering/boiling of water + safe storage. access to/use of basic sanitation in communities >75% basic sanitation coverage, HWWS after potential faecal contact)	828,651*	49,773,959*
Acute respiratory infections	0.13	0.08–0.16	CRA	plausible minimum risk (universal HWWS after potential faecal contact)	370,370	17,308,136
Protein-energy malnutrition	0.16*	0.15–0.17	based on diarrhoeal estimates	plausible minimum risk (see diarrhoea)	28,194*	2,995,329*
**total WASH-attributable disease burden using a plausible minimum risk**					**1,227,215**	**70,077,424**
Malaria	0.80	0.67–0.87	comparing universal safe water resource management (WRM) against no WRM	theoretical minimum risk (universal safe WRM)	354,924	29,707,805
Soil-transmitted helminth infections	1	1–1	burden completely WASH-attributed	theoretical minimum risk (universal safely managed water and sanitation, access to essential hygiene conditions and practice of essential hygiene behaviours)	6248	3,430,614
Trachoma	1	1–1	burden completely WASH-attributed	theoretical minimum risk (universal safely managed water and sanitation, access to essential hygiene conditions and practice of essential hygiene behaviours)	<10	244,471
**total WASH-attributable disease burden using a theoretical risk**					**361,175**	**33,382,890**

PAF: population attributable fraction, CI: confidence interval, DALYs: disability-adjusted life years, CRA: comparative risk assessment, HWWS: handwashing with soap, theoretical minimum risk: use of safely managed water and sanitation services, access to essential hygiene conditions and practice of essential hygiene behaviour, plausible minimum risk: boiling/filtering of drinking water with subsequent safe storage, access to/use of basic sanitation in a community with >75% basic sanitation coverage, handwashing with soap after potential faecal contact, feasible minimum risk: access to/use of basic drinking water and basic sanitation services, disease burden estimates are for low- and middle-income countries, diarrhoea and acute respiratory infections include disease burden in high-income countries from inadequate hygiene.

#### 3.3.2 Protein-energy malnutrition

Combining the fraction of diarrhoeal disease burden attributed to inadequate WASH in children below five years of age (adjusted estimate) with the estimate of 25% of stunting attributable to repeated diarrhoea episodes by country (Checkley et al., 2008) resulted in the attribution of 16% of malnutrition to inadequate water, sanitation and hygiene for 2016 (Table 10). These estimates do not include the consequences of protein-energy malnutrition on other diseases and associated mortality. WASH-attributable disease burden from protein-energy malnutrition by region is given in Table S7 in the Supplementary File 1.

Using non-adjusted diarrhoea estimates to calculate the WASH-attributable protein-energy malnutrition burden resulted in the attribution of 20% of malnutrition to inadequate WASH and in 34,000 WASH-attributable deaths in children below five years of age (Supplementary File S1, Table S8).

#### 3.3.3 Schistosomiasis

Using the available exposure and exposure-response information, it is estimated that 43% or 10,400 deaths could have been prevented by improving drinking water and sanitation services in 2016 (Table 10). Inadequate drinking water is responsible for 5700 deaths and inadequate sanitation for 6300 deaths. WASH-attributable disease burden from schistosomiasis by region is given in Table S9 in the Supplementary File 1.

The sensitivity analysis using the previously estimated PAF of 82% based on expert survey (Prüss-Ustün et al., 2016) would result in about 20,000 WASH-attributable Schistosomiasis deaths.

#### 3.3.4 Malaria

It is estimated that 80% of malaria was attributable to non-existent water resource management which resulted in 355,000 WASH-attributable malaria deaths in 2016 (Table 10).

A sensitivity analysis using previously estimated regional PAFs for malaria that were based on expert survey (Prüss-Ustün et al., 2016) resulted in 187,000 WASH-attributable malaria deaths in 2016.

#### 3.3.5 Soil-transmitted helminth infections and trachoma

Assuming 100% of soil-transmitted helminth infections and trachoma cases are attributable to inadequate WASH, over 6000 deaths could have been prevented in 2016 through safely managed water and sanitation, access to essential hygiene conditions and practice of essential hygiene behaviours (Table 10).

WASH-attributable disease burden estimates (in deaths and DALYs) by country and health outcome is detailed in Supplementary Files S4 (deaths) and S5 (DALYs).

## 4 Discussion

It is estimated that 1.6 million deaths and 105 million DALYs are attributable to inadequate WASH, including only diseases which could be quantified, representing 2.8% of total deaths and 3.9% of total DALYs in 2016. Of those, 829,000 deaths are due to diarrhoeal disease. Sixty per cent of the overall diarrhoea burden, 13% of the burden from acute respiratory infections, 16% of the burden of protein-energy malnutrition, 43% of the schistosomiasis burden, 80% of the malaria burden and 100% of both the burden from soil-transmitted helminth infections and trachoma burden are attributed to inadequate WASH.

### 4.1 Discussion of results

Compared to our previous burden of diarrhoeal disease assessment for the year 2012 (Prüss-Ustün et al., 2014), we now attribute about 17,000 less deaths to inadequate water (2012: 502,000 deaths, 2016: 485,000 deaths), 152,000 additional deaths to inadequate sanitation (2012: 280,000 deaths, 2016: 432,000 deaths) and 132,000 less deaths to inadequate hygiene behaviours (2012: 297,000 deaths, 2016: 165,000 deaths). Especially the methods for exposure assessment of both inadequate sanitation and inadequate hygiene behaviours have been revised using updated evidence. The consideration of health impacts from poor sanitation coverage in the community led to a significant increase of disease burden from inadequate sanitation. Furthermore, we are no longer relying on observations of handwashing frequency which are usually not nationally representative. Diarrhoea deaths attributable to inadequate WASH also changed due to reductions in overall diarrhoeal mortality (WHO, 2018a) and updated exposure-response relationships (Wolf et al., 2018a).

For comparison with similar estimates, the comparative risk assessment for the year 2016 for the Global Burden of Disease Study conducted by the Institute for Health Metrics and Evaluation attributed 89% of diarrhoea deaths and 8% of deaths from acute respiratory infections to inadequate WASH (Gakidou et al., 2017) – compared to 60% and 13% in this assessment. Differences compared to our estimates are mainly due to our approach of adjusting some WASH interventions for non-blinding bias (only diarrhoeal disease burden estimates, see discussion below), different approaches of exposure assessment and different minimum risk exposure (counterfactual) levels. The Institute for Health Metrics and Evaluation considers sewered sanitation as the sanitation counterfactual, which is however not necessarily supported by recent evidence nor for rural areas (Baum et al., 2013; WHO and UNICEF, 2017). Community sanitation coverage is not taken into account and availability of basic handwashing facilities is used as exposure parameter which does not match the parameter of the exposure-response relationship which is handwashing with soap at times of potential pathogen exposure.

Recent WASH disease burden estimates have varied considerably: in 2010 the Global Burden of Disease Study estimated 337,000 deaths from inadequate WASH (Lim et al., 2012) while subsequently reporting 1,399,000 deaths in 2013 (Forouzanfar et al., 2015), 1,766,000 deaths in 2015 (Forouzanfar et al., 2016), 1,661,000 deaths in 2016 (Gakidou et al., 2017) and 1,610,000 in 2017 (Stanaway et al., 2018). The initial increase was mainly due to the fact that the first counterfactuals for estimating WASH-attributable burden of disease were improved drinking water sources and improved sanitation facilities as defined by the JMP (WHO and UNICEF, undated). Improved drinking water sources are often unreliable and of poor water quality while improved sanitation is often not safely managed and does not protect the community (Bain et al., 2014; Clasen et al., 2014; WHO and UNICEF, 2017). More recent WASH-attributable global burden of disease assessments recognize health impacts from improvements in drinking water and sanitation beyond improved water sources and sanitation facilities, i.e., piped water sources, household water treatment and sewered sanitation, and from considering personal hygiene as separate risk factor. Since the 2015 assessment, more diseases have been added in the Global Burden of Disease assessments such as typhoid and paratyphoid fever in 2015 (Forouzanfar et al., 2016) and acute respiratory infections in 2016 and 2017 (Gakidou et al., 2017; Stanaway et al., 2018).

The positive side of a high WASH-attributable disease burden is the great potential for disease burden reduction. In theory, the entire estimated disease burden could have been prevented through interventions. These interventions vary depending on the health outcome and the chosen counterfactual exposure distribution. Diarrhoea, acute respiratory infections, malnutrition and schistosomiasis will require improvements of drinking water and sanitation services and increased handwashing with soap. The same is true for soil-transmitted helminth infections and trachoma, however to completely prevent these infections more radical and comprehensive WASH interventions are required (safely managed drinking water and sanitation services, access to essential hygiene conditions and practice of essential hygiene behaviours). Additionally, the prevention of soil-transmitted helminth infections might require the proper treatment of human waste and adequate food hygiene to prevent infections that occur through the use of human faeces as fertilizer (Anuar et al., 2014; Strunz et al., 2014). Trachoma prevention might include the need for a stronger emphasis on comprehensive hygiene practices including facewashing (Stocks et al., 2014). Finally to reduce the WASH-attributable malaria disease burden, interventions will be required that lead to environmental modification and manipulation, including water resource management as main component, and changes of the human habitat, including siting of settlements away from breeding sites (Keiser et al., 2005).

### 4.2 Limitations

This WASH-attributable burden of disease assessment is limited to some selected diseases and adverse health outcomes and does not take into account a large amount of other adverse health outcomes (examples are given in Table 1) that are at least partly WASH-attributable and that could be prevented through improved WASH management. Additionally, the here presented estimates do not capture disease burden from, for example, water-borne disease outbreaks, flooding and droughts or disease burden in certain populations such as refugees, internally displaced persons, and the homeless or certain exposure settings such as healthcare facilities, schools, workplaces and other public places. Additionally, adequate WASH and treatment of wastewater (from households, intensive livestock raising and industry) can reduce environmental drivers of antimicrobial resistance (Bürgmann et al., 2018; O'Neill, 2016; WHO, 2014), an increasingly serious threat to global public health (WHO, 2018g). WASH-attributable disease burden estimates refer predominantly to LMICs as most of the epidemiological evidence originates from these countries.

This analysis considers WASH-attributable deaths and DALYs from a range of diseases and conditions including diarrhoea, acute respiratory infections, protein-energy malnutrition, schistosomiasis, malaria, soil-transmitted helminth infections and trachoma. Some WASH-attributable disease burden estimates, i.e., for diarrhoea and respiratory infections, are based on CRA and the exposure-response relationship on meta-analysis of intervention studies. The remaining diseases have been estimated using more limited exposure or exposure-response information which required more assumptions. WASH-attributable disease burden estimates for the latter diseases include therefore greater uncertainties. The WASH-attributable estimates of the burden of respiratory infections are calculated using a dose-response relationship from intervention studies not adjusted for likely bias due to non-blinding. The malnutrition estimates are based on the diarrhoea estimates and therefore omit other pathways through which WASH can have an impact on malnutrition such as subclinical enteric infections and environmental enteropathy (Rogawski and Guerrant, 2017). In addition, these estimates include only stunting and omit other forms of malnutrition such as underweight and wasting. Stunting, compared to wasting and underweight, is the more severe form of malnutrition, is associated with chronic and recurrent undernutrition, e.g., from frequent infectious disease, and prevents children from reaching their physical and cognitive potential (WHO, 2018h). There is usually considerable overlap between stunting, wasting and underweight (Myatt et al., 2018). The estimate of the fraction of WASH-attributable stunting is based on the fraction of stunting attributable to repeated diarrhoea episodes (Checkley et al., 2008) which is combined with the fraction of WASH-attributable diarrhoea. In young children from low-income countries (where the bulk of the global burden of diarrhoea occurs) repeated diarrhoea episodes are the norm: e.g., children under three years old experience on average three episodes of diarrhoea every year (WHO, 2017b). Recent findings from the GEMS study suggested that children with both moderate/severe and less-severe diarrhoea had a significantly increased risk for stunting (Kotloff et al., 2019). Global health estimates for diarrhoeal disease burden which are used for WASH-attributable disease burden estimation can be subject to considerable under-reporting, especially for countries without well-functioning death registration systems for which estimates rely heavily on surveys and censuses (WHO, 2018i).Our estimate of 16% of malnutrition is broadly consistent with a Cochrane review that concluded that WASH interventions might have a small benefit on length growth (Dangour et al., 2013). The schistosomiasis exposure-response function is based on observational studies only (Freeman et al., 2017; Grimes et al., 2014) and the counterfactual exposure distribution is use of basic water and sanitation services which represents a feasible minimum risk exposure distribution only. The counterfactual exposure distribution for malaria – universal exposure to safe water resource management (Keiser et al., 2005) – differs from the exposure distributions of the other diseases which are related to the use of certain WASH services. From the above it can be concluded that our disease burden estimates are likely underestimating the true disease burden of inadequate WASH.

While some have argued that the counterfactual exposure distribution used for risk factor-attributable disease burden estimation should represent what can be achieved through interventions (Greenland, 2002; Steenland and Armstrong, 2006), others advocate the use of multiple exposure distributions including those which might not be achievable by currently available interventions to appreciate the size of the problem (Murray et al., 2003). Based on the available evidence – especially regarding the exposure-response relationship – our WASH-attributable disease burden estimates are based on different – including feasible, plausible and theoretical minimum risk – counterfactual definitions. Especially the feasible (only used for schistosomiasis) but also the plausible minimum risk exposure levels represent interim levels on which further improvements are possible and necessary. These interim exposure levels should be replaced with the theoretical minimum risk exposure distribution of safely managed water and sanitation, access to essential hygiene conditions and practice of essential hygiene behaviours when the available evidence allows this. The JMP currently provides country-level data for access to safely managed drinking water and sanitation services only for a limited number of countries (WHO and UNICEF, 2018b). In addition, there is to date no matching exposure-response relationship from meta-analysis between safely managed drinking water or sanitation and disease outcome. Even the theoretical minimum risk exposure distribution might underestimate the true WASH-attributable disease burden which is supported by evidence of residual WASH-attributable diarrhoea burden in high-income countries (Gunnarsdottir et al., 2012; Setty et al., 2017). Evidence on health impacts of Water Safety Plans which are implemented increasingly throughout the world (WHO and IWA, 2017) could potentially strengthen the theoretical minimum risk exposure distribution for burden of disease assessment and add estimates for high-income countries in the future (Gunnarsdottir et al., 2012; Setty et al., 2017). Exposure levels do also not include bottled or packaged water which is used increasingly in many countries (statista, 2016). Bottled water was frequently shown to be of high microbial quality (Bain et al., 2014; Fisher et al., 2015; UNICEF and WHO, 2015; Williams et al., 2015; Wright et al., 2016) and was associated with a decreased risk for diarrhoea compared to piped water (Sima et al., 2012). Both country-level exposure data and the matching exposure-response relationship between bottled water use and health outcome are currently lacking. Changing from a feasible or plausible minimum risk exposure level to a theoretical minimum risk exposure level as the counterfactual for WASH-attributable disease burden estimation (relevant for diarrhoea, acute respiratory infections, malnutrition, and schistosomiasis) might considerably increase WASH-attributable disease burden estimates. This is supported by historical evidence of large reductions of child and overall mortality following improvements towards safely managed water and sanitation infrastructure in high-income countries (Alsan and Goldin, 2018; Bell and Millward, 1998; Cutler et al., 2006).

The WASH-attributable burden of disease assessment from most included diseases is based on WASH interventions, many of which were poorly implemented, had low compliance and promoted or installed technologies with disputable effectiveness. Therefore, the estimated WASH-attributable disease fractions can be interpreted as estimates of the fractions of disease preventable through implementing these interventions. We do adjust the diarrhoeal disease burden estimates for the likely overestimation of health impacts due to non-blinding by adjusting the results of each non-blinded point-of-use drinking water and hygiene intervention (Wolf et al., 2018a, 2014). This approach down-weights biased studies and – in our case – results in reduced estimated health impacts. The above cited issues on poor WASH interventions are however likely to underestimate the disease burden attributable to inadequate WASH. This is one more reason why our assessment assures conservative estimates which are at the lower end of the assumed truth. The WASH-attributable disease burden estimates from diarrhoea, soil-transmitted helminth infections and protein-energy malnutrition have undergone country consultations which ensure the use of all available and eligible exposure and disease data and compatible data categories.

The formula combining disease burden estimates from water, sanitation and hygiene (eq. (2)) assumes that risk factors are independent (Steenland and Armstrong, 2006). This assumption is likely to be an oversimplification for WASH as, for instance, handwashing promotion is unlikely to be effective if water quantity is limited. However, this approach has been applied in the assessment for ease of interpretation of the results, and in the absence of a more suitable approach.

WASH-attributable morbidity for some diseases in our analysis (diarrhoea, schistosomiasis) is estimated separately for the different components of WASH (water, sanitation and hygiene are analysed in three separate models). This approach ignores that the different WASH components affect disease in conjunction. The meta-regression model (Wolf et al., 2018a) that was used to generate the exposure-response relationships between WASH and diarrhoea, however adjusted for baseline WASH of the other categories and included further covariates. A multi-risk model might nevertheless be the preferred approach for WASH-attributable disease burden assessment in the future. Including all three WASH components in one model would also take account of the fact that the three risk factors (inadequate water, inadequate sanitation and inadequate hygiene) are often likely to vary simultaneously, e.g. improving access to or use of water facilities might improve hygiene behaviours and sanitation at the same time.

The here presented WASH-attributable burden of disease estimates required different assumptions. We show through different sensitivity analyses that disease burden estimates can change by as much as a factor of two depending on assumptions, applied exposure-response relationships and counterfactual definitions. Especially the WASH-attributable schistosomiasis disease burden estimates, generated using the feasible minimum risk exposure distribution, are likely to be underestimated. Accordingly, estimates based on expert survey were considerably higher. Care should be taken to consider the approximate nature of the estimates which are however suitable to gauge the size of the problem, to compare the relative importance of diseases and risk factors and to monitor changes over time.

The attributable burden signifies the reduction in current or future disease burden if past exposure to a risk factor had been equal to the counterfactual exposure distribution (Murray et al., 2003). An assumption that is made when stating the PAF is that the formerly exposed group immediately attains disease risk of the unexposed group after removal or reduction of the exposure (Kowall and Stang, 2018; Rockhill et al., 1998). This is often not the case and additionally differs between different health outcomes. For example, diarrhoea disease reduction is likely to happen more immediate than changes in nutritional status, universal water resource management may take a considerable time to implement but once it is established disruption of mosquito habitats will probably follow quite quickly. These different time lags that are not apparent from the PAF need to be considered and are important for interpreting results, prevention efforts, research and policy.

## 5 Conclusions

An important fraction of overall deaths and DALYs in low- and middle-income countries is attributable to inadequate WASH. Burden of disease estimates have an approximate nature as they do not capture the complete list of WASH-attributable adverse health outcomes, exposed settings and populations and are dependent on assumptions, exposure-response functions and chosen counterfactual definitions that are often still based on imperfect WASH interventions.

To improve estimates of health benefits from WASH there is a need for well-designed trials that evaluate the effectiveness of safely managed water and sanitation services, access to essential hygiene conditions and practice of essential hygiene behaviours that reach high coverage and use in the communities. To improve health outcomes there is a strong need for research on implementation systems, intervention quality and intermediate outcomes such as exposure to faecal pathogens in the community. Additionally, data from high-income countries on WASH exposure distributions and exposure-response relationships might strengthen future definitions of the theoretical minimum exposure distribution and might enable more comprehensive WASH disease burden assessments.

## Appendix A Supplementary data

Supplementary File S1: Adjustment for non-blinding bias of interventions for exposure-response estimation between WASH and selected data tables of exposure-response relationships and disease burden estimates.

Supplementary File S2: WASH exposure data 2016.

Supplementary File S3: GATHER-checklist.

Supplementary File S4: Deaths attributable to inadequate WASH by cause and WHO member state for the year 2016.

Supplementary File S5: DALYs attributable to inadequate WASH by cause and WHO member state for the year 2016.
