# Supplementary material for: Burden of disease from inadequate water, sanitation and hygiene for selected adverse health outcomes: An updated analysis with a focus on low- and middle-income countries
Source: Int J Hyg Environ Health. 2019 Jun;222(5):765–77. doi: 10.1016/j.ijheh.2019.05.004 (PMC6593152; doi:10.1016/j.ijheh.2019.05.004)
Supplement: Multimedia component 1 [file mmc1.docx]

# Supplementary File 1: Adjustment for non-blinding bias of interventions for exposure-response estimation between WASH and diarrhoeal disease and diarrhoea and protein-energy malnutrition estimates unadjusted for non-blinding bias

Much of the description of the approach for adjusting certain WASH interventions for non-blinding bias is based on the explanations included in the systematic reviews and meta-analyses that generated the exposure-response relationships between WASH and diarrhoeal disease (1,2). Most WaSH interventions are unblinded and diarrhoea is self-reported in most intervention evaluation studies which may lead to biased reports of diarrhoea (3,4). We performed an additional analysis which incorporates bias adjustments for the point-of-use water quality and hygiene promotion interventions based on empirical evidence (3). These two types of WASH interventions were chosen for bias adjustment as these interventions usually aim exclusively to improve health which is apparent to the recipient. In contrast, water and sanitation interventions that improve supply are often less apparent to the recipient and have aims beyond health such as community development, environmental hygiene benefits and time savings of water collection. We therefore hypothesize that in POU water quality and hygiene interventions recipients are more prone to reporting bias than in water and sanitation supply interventions. More information and computational details are provided in the systematic reviews (1,2).

For the bias adjustment, a Bayesian meta-regression analysis was performed by subtracting a bias factor from the log risk ratio from each non-blinded study. This bias factor is based on 234 meta-analyses including a total of 1970 trials across a broad range of clinical areas, settings and types of experimental interventions including curative and preventive interventions (3). The bias factor reflects findings from the BRANDO meta-epidemiological study, which examined the distribution of bias apparently due to lack of blinding (3). For subjective outcomes (like self-report of diarrhoea), a ratio of odds ratios (ROR) of 0.78 (95% credible interval 0.65 to 0.92) is observed, which reflects a mean bias across the meta-analyses they studied. Specifically, it implies that odds ratios are smaller in non-blinded studies than they are in blinded studies. On the log(ROR) scale, this equates to a mean bias of −0.25 (−0.43 to −0.08), implying a variance of 0.00785. In addition, variability of biases across trials within a meta-analysis (SD 0.37, variance 0.1369) and the variability of biases across meta-analyses (SD 0.23; variance 0.0529) was estimated (3). On the basis of these findings, a prior based on the mean and all variation was chosen to best reflect the findings of the meta-epidemiological study and to adjust non-blinded household-level drinking-water and hygiene promotion interventions. This prior takes prior distribution Normal(−0.25, 0.2). The full variance is the sum of the variance components: 0.00785 + 0.1369 + 0.0529 = 0.2. It adjusts the biased studies and down-weights them considerably.

Table S1: Exposure-response relationship (risk ratios) for drinking water and diarrhoeal disease, (a) not adjusted for non-blinding, (b) adjusted for non-blinding in point-of-use filter treatment interventions

|  | Improved water exposure level | | |
| --- | --- | --- | --- |
|  | Basic drinking water, non-piped | Basic drinking water, piped water on premises | Point-of-use filter treatment + safe storage |
| Baseline water |  |  |  |
| (a) not adjusted for non-blinding |  |  |  |
| Surface water, unimproved or limited drinking water | 0.89 (0.77, 1.02) | 0.77 (0.64, 0.93) | 0.39 (0.32, 0.48) |
| Basic drinking water, non-piped | → | 0.87 (0.72,1.04) | 0.44 (0.34, 0.56) |
| Basic drinking water, piped water on premises | → | → | 0.50 (0.38, 0.66) |
| (b) adjusted for non-blinding |  |  |  |
| Surface water, unimproved or limited drinking water | 0.89 (0.77, 1.02) | 0.77 (0.64, 0.92) | 0.52 (0.35, 0.77) |
| Basic drinking water, non-piped | → | 0.87 (0.72,1.04) | 0.59 (0.39, 0.88) |
| Basic drinking water, piped water on premises | → | → | 0.68 (0.44,1.04) |

As the exposure-response relationships are based on intervention studies, “baseline water” refers to the drinking water at baseline or in the control group, and “improved water exposure level” refers to the drinking water post-intervention in the intervention group. Table adapted from Wolf et al. (2018a), results are adjusted for provision of safe water storage (a) (0.79 (0.64, 0.98)), (b) (0.87 (0.69, 1.11) and combined (with other WASH interventions) intervention: (a) (0.84 (0.70, 1.00)), (b) (0.85 (0.72, 1.01)). Exposure-response relationships and confidence intervals are calculated on the log-scale, confidence intervals after transformation are therefore not symmetric.

Table S2: Exposure-response relationship (risk ratios) for sanitation and hygiene and diarrhoeal disease, (a) not adjusted for non-blinding, (b) adjusted for non-blinding in hygiene interventions

|  | Improved sanitation exposure level |  |
| --- | --- | --- |
|  | Basic sanitation, community coverage ≤75% | Basic sanitation, community coverage >75% |
| Baseline sanitation |  |  |
| Open defection, unimproved or limited sanitation | 0.76 (0.51, 1.13) | 0.55 (0.34, 0.91) |
|  | **Improved hygiene exposure level** |  |
| Baseline hygiene | Handwashing with soap |  |
| (a) not adjusted for non-blinding |  |  |
| No handwashing with soap | 0.67 (0.59, 0.76) |  |
| (b) adjusted for non-blinding |  |  |
| No handwashing with soap | 0.86 (0.35, 2.07) |  |

As the exposure-response relationships are based on intervention studies “Baseline sanitation” and “Baseline hygiene” refer to sanitation and hygiene at baseline or in the control group and “Improved sanitation exposure level” and “Improved hygiene exposure level” refer to sanitation and hygiene post-intervention in the intervention group. Risk ratios taken from Wolf et al. (2018a). Exposure-response relationships and confidence intervals are calculated on the log-scale, confidence intervals after transformation are therefore not symmetric.

Table S3: Diarrhoea burden attributable to inadequate water by region, 2016 – unadjusted estimates

| Region | PAF | (95% CI) | Deaths | (95% CI) | DALYs  (in 1 000s) | (95% CI) |
| --- | --- | --- | --- | --- | --- | --- |
| Sub-Saharan Africa, LMIC, | 0.55 | (0.48-0.60) | 352,459 | (306,808-386,511) | 22,885 | (19,934-25,083) |
| America, LMICs | 0.43 | (0.31-0.51) | 9,723 | (6,965-11,596) | 792 | (562-949) |
| Eastern Mediterranean, LMICs | 0.54 | (0.46-0.60) | 68,287 | (58,072-75,642) | 5,143 | (4,370-5,700) |
| Europe, LMICs | 0.32 | (0.23-0.39) | 1,561 | (1,109-1,889) | 222 | (156-270) |
| South-East Asia, LMICs | 0.45 | (0.37-0.51) | 237,988 | (193,102-270,914) | 11,330 | (9,196-12,898) |
| Western Pacific, LMICs | 0.33 | (0.26-0.39) | 9,029 | (7,008-10,591) | 782 | (598-925) |
| Total LMICs | 0.50 | (0.43-0.56) | 679,047 | (578,151-754,234) | 41,154 | (35,189-45,626) |

DALYs: disability-adjusted life years, PAF: population-attributable fraction; LMICs: low- and middle-income countries.

Table S4: Diarrhoea burden attributable to inadequate hand hygiene by region, 2016 – unadjusted estimates

| Region | PAF | | (95% CI) | | Deaths | | (95% CI) | | DALYs  (in 1 000s) | | (95% CI) | |
| --- | --- | --- | --- | --- | --- | --- | --- | --- | --- | --- | --- | --- |
| Sub-Saharan Africa, all | | 0.31 | | (0.22-0.38) | | 200,545 | | (140,629-247,079) | | 12,988 | | (9,120-15,994) |
| America, LMICs | | 0.24 | | (0.17-0.31) | | 5,543 | | (3,788-6,974) | | 455 | | (310-573) |
| America, HICs | | 0.20 | | (0.11-0.28) | | 2,403 | | (1,396-3,408) | | 63 | | (37-91) |
| Eastern Mediterranean, LMICs | | 0.29 | | (0.19-0.37) | | 36,137 | | (23,678-46,823) | | 2,724 | | (1,805-3,513) |
| Eastern Mediterranean, HICs | | 0.20 | | (0.11-0.28) | | 89 | | (51-126) | | 13 | | (8-18) |
| Europe, LMICs | | 0.27 | | (0.18-0.35) | | 1,308 | | (860-1,663) | | 175 | | (115-224) |
| Europe, HICs | | 0.20 | | (0.13-0.26) | | 3,140 | | (2,025-4,130) | | 74 | | (48-97) |
| South-East Asia, all | | 0.26 | | (0.18-0.33) | | 138,323 | | (95,395-173,629) | | 6,512 | | (4,491-8,176) |
| Western Pacific, LMICs | | 0.29 | | (0.21-0.36) | | 8,013 | | (5,626-9,931) | | 713 | | (500-883) |
| Western Pacific, HICs | | 0.20 | | (0.12-0.27) | | 801 | | (476-1,113) | | 15 | | (9-21) |
| Total | | 0.29 | | (0.20-0.35) | | 396,301 | | (276,895-490,531) | | 23,733 | | (16,596-29,354) |

DALYs: disability-adjusted life years, PAF: population-attributable fraction; LMICs: low- and middle-income countries, HICs: high-income countries.

Table S5: Diarrhoea burden attributable to the cluster of inadequate water, sanitation and hand hygiene by region, 2016 – unadjusted estimates

| Region | PAF | | (95% CI) | | Deaths | | (95% CI) | | DALYs  (in 1 000s) | | (95% CI) | |
| --- | --- | --- | --- | --- | --- | --- | --- | --- | --- | --- | --- | --- |
| Sub-Saharan Africa, all | | 0.80 | | (0.79-0.82) | | 516,002 | | (506,846-524,424) | | 33,447 | | (32,848-33,995) |
| America, LMICs | | 0.62 | | (0.59-0.66) | | 14,192 | | (13,456-14,905) | | 1,158 | | (1,093-1,222) |
| America, HICs | | 0.20 | | (0.12-0.27) | | 2,403 | | (1,396-3,408) | | 63 | | (37-91) |
| Eastern Mediterranean, LMICs | | 0.76 | | (0.72-0.79) | | 95,979 | | (91,503-100,055) | | 7,218 | | (6,906-7,504) |
| Eastern Mediterranean, HICs | | 0.20 | | (0.12-0.27) | | 89 | | (51-126) | | 13 | | (8-18) |
| Europe, LMICs | | 0.52 | | (0.48-0.56) | | 2,507 | | (2,316-2,691) | | 346 | | (319-371) |
| Europe, HICs | | 0.20 | | (0.15-0.24) | | 3,140 | | (2,025-4,130) | | 74 | | (48-97) |
| South-East Asia, all | | 0.71 | | (0.67-0.75) | | 373,613 | | (351,777-393,381) | | 17,681 | | (16,636-18,629) |
| Western Pacific, LMICs | | 0.61 | | (0.57-0.64) | | 16,620 | | (15,665-17,532) | | 1,456 | | (1,364-1,544) |
| Western Pacific, HICs | | 0.20 | | (0.13-0.26) | | 801 | | (476-1,113) | | 15 | | (9-21) |
| Total | | 0.74 | | (0.72-0.76) | | 1,025,346 | | (1,001,945-1,047,842) | | 61,472 | | (60,246-62,657) |

DALYs: disability-adjusted life years, PAF: population-attributable fraction; LMICs: low- and middle-income countries, HICs: high-income countries.

Table S6: Burden of acute respiratory infections attributable to inadequate hygiene behaviours by region, 2016

| Region | PAF | (95% CI) | Deaths | (95% CI) | DALYs  (in 1 000s) | (95% CI) |
| --- | --- | --- | --- | --- | --- | --- |
| Sub-Saharan Africa, all | 0.15 | (0.10-0.19) | 134,199 | (89,077-175,966) | 8,625 | (5,731-11,293) |
| America, LMICs | 0.11 | (0.07-0.14) | 25,022 | (16,371-33,059) | 683 | (447-898) |
| America, HICs | 0.09 | (0.05-0.13) | 6,847 | (3,702-10,251) | 128 | (68-193) |
| Eastern Mediterranean, LMICs | 0.13 | (0.08-0.18) | 29,903 | (18,762-40,602) | 2,070 | (1,299-2,826) |
| Eastern Mediterranean, HICs | 0.09 | (0.05-0.13) | 702 | (393-1,047) | 21 | (12-31) |
| Europe, LMICs | 0.12 | (0.08-0.17) | 9,252 | (5,577-12,868) | 374 | (228-516) |
| Europe, HICs | 0.09 | (0.05-0.12) | 14,667 | (9,332-19,783) | 195 | (124-262) |
| South-East Asia, all | 0.12 | (0.08-0.16) | 94,304 | (62,061-124,266) | 3,775 | (2,483-4,974) |
| Western Pacific, LMICs | 0.14 | (0.09-0.18) | 40,802 | (27,158-53,196) | 1,266 | (843-1,650) |
| Western Pacific, HICs | 0.09 | (0.05-0.13) | 14,672 | (7,997-21,847) | 172 | (95-254) |
| Total | 0.13 | (0.08-0.16) | 370,365 | (245,444-484,900) | 17,308 | (11,445-22,635) |

DALYs: disability-adjusted life years, PAF: population-attributable fraction; LMICs: low- and middle-income countries, HICs: high-income countries.

Table S7: Burden of protein-energy malnutrition in children below five years of age attributable to the cluster of inadequate water, sanitation and hygiene behaviours by region, 2016- based on adjusted diarrhoea estimates

| Region | PAF | (95% CI) | Deaths | (95% CI) | DALYs  (in 1 000s) | (95% CI) |
| --- | --- | --- | --- | --- | --- | --- |
| Sub-Saharan Africa, LMICs | 0.17 | (0.13-0.20) | 23,446 | (17,906- 28,174) | 2,212 | (1,689-2,658) |
| America, LMICs | 0.11 | (0.06-0.16) | 493 | (254-704) | 54 | (26-79) |
| Eastern Mediterranean, LMICs | 0.16 | (0.11-0.20) | 1,557 | (1,113-1,930) | 187 | (128-236) |
| Europe, LMICs | 0.07 | (0.01-0.13) | 21 | (3-39) | 6 | (1-11) |
| South-East Asia, LMICs | 0.15 | (0.10-0.18) | 2,394 | (1,689-3,016) | 499 | (352-630) |
| Western Pacific, LMICs | 0.12 | (0.06-0.17) | 282 | (150-397) | 37 | (19-54) |
| Total LMICs | 0.16 | (0.15-0.17) | 28,194 | (26,369-29,900) | 2,995 | (2,794-3,186) |

DALYs: disability-adjusted life years, PAF: population-attributable fraction; LMICs: low- and middle-income countries.

**Table S8: Burden of protein-energy malnutrition in children below five years of age attributable to the cluster of inadequate water, sanitation and hand hygiene by region, 2016- based on unadjusted diarrhroea estimates**

| Region | PAF | (95% CI) | Deaths | (95% CI) | DALYs  (in 1 000s) | (95% CI) |
| --- | --- | --- | --- | --- | --- | --- |
| Sub-Saharan Africa, LMICs | 0.20 | (0.19-0.21) | 28,011 | (26,514-29,371) | 2,642 | (2,501-2,770) |
| America, LMICs | 0.16 | (0.14-0.18) | 696 | (607-779) | 78 | (67-87) |
| Eastern Mediterranean, LMICs | 0.19 | (0.18-0.21) | 1,912 | (1,768-2,040) | 233 | (214-251) |
| Europe, LMICs | 0.12 | (0.10-0.15) | 36 | (28-44) | 11 | (8-13) |
| South-East Asia, LMICs | 0.18 | (0.17-0.20) | 3,008 | (2,782-3,218) | 629 | (581-673) |
| Western Pacific, LMICs | 0.16 | (0.15-0.18) | 392 | (352-429) | 53 | (47-58) |
| Total LMICs | 0.20 | (0.19-0.20) | 34,055 | (33,554-34,533) | 3,645 | (3,586-3,701) |

DALYs: disability-adjusted life years, PAF: population-attributable fraction; LMICs: low- and middle-income countries.

Table S9: Burden of schistosomiasis attributable to inadequate water and sanitation by region, 2016

| Region | PAF | (95% CI) | Deaths | (95% CI) | DALYs  (in 1 000s) | (95% CI) |
| --- | --- | --- | --- | --- | --- | --- |
| Sub-Saharan Africa, LMICs | 0.46 | (0.42-0.49) | 9,580 | (8,836- 10,292) | 1,017 | (943-1,088) |
| America, LMICs | 0.09 | (0.04-0.14) | 52 | (23-81) | 9 | (4-14) |
| Eastern Mediterranean, LMICs | 0.33 | (0.28-0.38) | 705 | (602-805) | 55 | (48-61) |
| Europe, LMICs | -- | -- | 0 | -- | 0 | -- |
| South-East Asia, LMICs | 0.21 | (0.13-0.30) | 5 | (3-7) | 0 | -- |
| Western Pacific, LMICs | 0.17 | (0.11-0.23) | 63 | (39-86) | 14 | (10-19) |
| Total LMICs | 0.43 | (0.40-0.46) | 10,405 | (9,643-11,108) | 1,096 | (1,020-1,166) |

DALYs: disability-adjusted life years, PAF: population-attributable fraction; LMICs: low- and middle-income countries.

# References

1. Wolf J, Hunter PR, Freeman MC, Cumming O, Clasen T, Bartram J, et al. Impact of Drinking Water, Sanitation and Hand Washing with Soap on Childhood Diarrhoeal Disease: Updated Meta-Analysis and –Regression. Trop Med Int Health. 2018;23(5).

2. Wolf J, Prüss-Ustün A, Cumming O, Bartram J, Bonjour S, Cairncross S, et al. Assessing the impact of drinking-water and sanitation on diarrhoeal disease in low-and middle-income settings: A systematic review and meta-regression. Trop Med Int Health. 2014;19(8):928–42.

3. Savović J, Jones HE, Altman DG, Harris RJ, Jüni P, Pildal J, et al. Influence of reported study design characteristics on intervention effect estimates from randomized, controlled trials. Ann Intern Med. 2012;157(6):429–438.

4. Wood L, Egger M, Gluud LL, Schulz KF, Jüni P, Altman DG, et al. Empirical evidence of bias in treatment effect estimates in controlled trials with different interventions and outcomes: meta-epidemiological study. BMJ. 2008;336(7644):601.
