# Supplementary material for: Burden of disease from inadequate water, sanitation and hygiene for selected adverse health outcomes: An updated analysis with a focus on low- and middle-income countries
Source: Int J Hyg Environ Health. 2019 Jun;222(5):765–77. doi: 10.1016/j.ijheh.2019.05.004 (PMC6593152; doi:10.1016/j.ijheh.2019.05.004)
Supplement: Multimedia component 3 [file mmc3.docx]

**
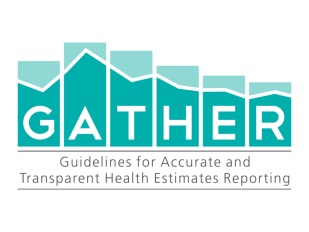
Checklist of information that should be included in new reports of global health estimates**

| Item # | Checklist item | Reported in the following section |
| --- | --- | --- |
| Objectives and funding | | |
| 1 | Define the indicator(s), populations (including age, sex, and geographic entities), and time period(s) for which estimates were made. | 🡪 methods: “Framework for estimation”, 1^st^ and 2^nd^ paragraph |
| 2 | List the funding sources for the work. | 🡪 financial disclosure information |
| Data Inputs | | |
| *For all data inputs from multiple sources that are synthesized as part of the study:* | | |
| 3 | Describe how the data were identified and how the data were accessed. | 🡪 methods: “Framework for estimation”, last sentence for data on disease burden (deaths and DALYs), for data on the exposure-risk relationships and the exposure estimates see sections of respective diseases |
| 4 | Specify the inclusion and exclusion criteria. Identify all ad-hoc exclusions. | 🡪 methods: “Framework for estimation”, 1^st^ and 2^nd^ paragraph |
| 5 | Provide information on all included data sources and their main characteristics. For each data source used, report reference information or contact name/institution, population represented, data collection method, year(s) of data collection, sex and age range, diagnostic criteria or measurement method, and sample size, as relevant. | 🡪 methods: “Framework for estimation”, last sentence for data on disease burden (deaths and DALYs), for data on the exposure-risk relationships and the exposure estimates see sections of respective diseases |
| 6 | Identify and describe any categories of input data that have potentially important biases (e.g., based on characteristics listed in item 5). | 🡪 methods: “Adjustment for non-blinding bias of interventions for exposure-response estimation” |
| *For data inputs that contribute to the analysis but were not synthesized as part of the study:* | | |
| 7 | Describe and give sources for any other data inputs. | NA (there are no other data sources than the ones already described) |
| *For all data inputs:* | | |
| 8 | Provide all data inputs in a file format from which data can be efficiently extracted (e.g., a spreadsheet rather than a PDF), including all relevant meta-data listed in item 5. For any data inputs that cannot be shared because of ethical or legal reasons, such as third-party ownership, provide a contact name or the name of the institution that retains the right to the data. | WHO data on overall burden by disease and age group is publicly available and the link to the respective website is given in the article (🡪 methods: “Framework for estimation”, last sentence;  exposure data on the different levels of water, sanitation and hygiene has been made available as a supplementary file (S3); the population attributable fractions (plus 95% Cis) are given in Table 14 |
| Data analysis | | |
| 9 | Provide a conceptual overview of the data analysis method. A diagram may be helpful. | 🡪 methods: “Population attributable fractions of disease for individual risk factors and for the cluster of risks”, “Population attributable fractions of disease for individual risk factors and for the cluster of risks”, “Estimation of burden of disease attributable to inadequate WASH”, and “Uncertainty estimates”, Figures 1 and 2 |
| 10 | Provide a detailed description of all steps of the analysis, including mathematical formulae. This description should cover, as relevant, data cleaning, data pre-processing, data adjustments and weighting of data sources, and mathematical or statistical model(s). | 🡪 methods: “Population attributable fractions of disease for individual risk factors and for the cluster of risks”, “Population attributable fractions of disease for individual risk factors and for the cluster of risks”, “Estimation of burden of disease attributable to inadequate WASH”, and “Uncertainty estimates” |
| 11 | Describe how candidate models were evaluated and how the final model(s) were selected. | 🡪 methods: “Choice of counterfactual exposure levels for WASH-attributable disease burden estimation” |
| 12 | Provide the results of an evaluation of model performance, if done, as well as the results of any relevant sensitivity analysis. | Results of sensitivity analyses are presented in the results section under the respective disease headings and in the supplementary file. |
| 13 | Describe methods for calculating uncertainty of the estimates. State which sources of uncertainty were, and were not, accounted for in the uncertainty analysis. | 🡪 methods: “Uncertainty estimates” |
| 14 | State how analytic or statistical source code used to generate estimates can be accessed. | NA |
| Results and Discussion | | |
| 15 | Provide published estimates in a file format from which data can be efficiently extracted. | 🡪 results: Tables 7-14, tables in supplementary file 1, supplementary files 2, 4 and 5. |
| 16 | Report a quantitative measure of the uncertainty of the estimates (e.g. uncertainty intervals). | 🡪 results: Tables 7-14, tables in supplementary file 1, supplementary files 2, 4 and 5, all estimates are accompanied by confidence intervals |
| 17 | Interpret results in light of existing evidence. If updating a previous set of estimates, describe the reasons for changes in estimates. | 🡪discussion: “Discussion of results” |
| 18 | Discuss limitations of the estimates. Include a discussion of any modelling assumptions or data limitations that affect interpretation of the estimates. | 🡪 discussion: “Limitations” |

*This checklist should be used in conjunction with the GATHER statement and Explanation and Elaboration document, found on gather-statement.org*
